# Spontaneous abortion as differential diagnosis of intermittent glomerular proteinuria in inactive systemic lupus erythematosus

**DOI:** 10.1002/ccr3.7686

**Published:** 2023-07-20

**Authors:** Fabian T. H. Ullrich, Alla Skapenko, Hendrik Schulze‐Koops

**Affiliations:** ^1^ Division of Rheumatology and Clinical Immunology, Department of Medicine IV, LMU University Hospital LMU Munich Munich Germany

**Keywords:** abortion, pregnancy, proteinuria, renal lupus, systemic lupus erythematosus

## Abstract

In women of childbearing age, severe proteinuria in systemic lupus erythematosus raises concern for renal involvement and pregnancy complications. While persisting renal loss of protein is known to culminate in extensive interventions, intermittent proteinuria in inactive disease requires an adjusted approach. Contextual awareness of this urinary finding is thus essential.

## Graphical abstract

Diagnostic algorithm of intermittent glomerular proteinuria in pregnant patients with SLE.

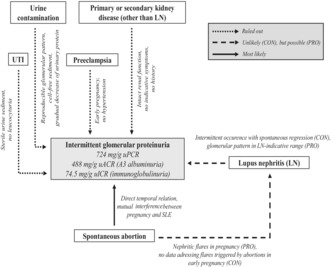

## 1 INTRODUCTION

Normal urinary protein excretion is <150 mg daily with <30 mg albumine, while during gestation up to 300 mg protein is considered physiological.^1^ One feasible method of quantifying protein excretion is the calculation of the urine protein‐/albumine‐to‐creatinine‐ratio (uPCR/uACR). According to the 2012 Kidney Disease: Improving Global Outcomes (KDIGO)‐practice guidelines, albuminuria is divided into three classes: A1 (<30 mg/g uACR, *normal to mildly increased*), A2 (30–300 mg/g uACR, *moderately increased*), and A3 (>300 mg/g uACR, *severely increased*).^2^ In glomerular (nonselective) proteinuria, in addition to albumine, high‐molecular‐mass proteins like immunoglobulins appear in the urine.^3^ In systemic lupus erythematosus (SLE), new‐onset proteinuria and albuminuria is indicative for lupus nephritis (LN), even when occurring isolated and subnephrotic.^4^, ^5^ Differential diagnoses include further disorders, such as pregnancy complications, which are not necessarily lupus‐related. Distinguishing renal disease from pregnancy‐related complications in SLE is difficult but important, because SLE is associated with adverse maternal and fetal events, while pregnancy is also a cause of disease relapses.^6^, ^7^ Common to all causes of pathological proteinuria in SLE is that they are related to disease activity and thus require therapeutic intervention, as proteinuria does not normalize on its own. We report a case of intermittent, significant proteinuria and albuminuria of glomerular origin directly related to a miscarriage in a clinically and serologically inactive SLE patient.

## 2 CASE PRESENTATION

### 2.1 SLE history

A 39‐year‐old female with SLE presented at our clinic for a routine checkup in late 2022. Diagnosis of SLE was made in 2009 with clinical (Raynaud's syndrome, fatigue, and photosensitivity) and humoral (anti‐nuclear antibodies, anti‐dsDNA‐antibodies, lupus anticoagulant [LAC], C3 complement consumption, and thrombocytopenia) criteria. Due to stable disease, medication from 2009 to the current admission consisted only of monotherapy with hydroxychloroquine in weight‐adjusted dosing of 5 mg/kg/day of actual body weight. The patient adhered to the therapy without interruptions. Stable low‐grade thrombocytopenia was the only relevant abnormality in recent years and the patient was clinically asymptomatic without internal organ involvement. Absolute platelet counts (APC) undulated between 70 and 140 G/L since diagnosis was made. To that date, systemic lupus erythematosus disease activity index 2000 (SLEDAI‐2K) during most visits had a score of 0, indicating inactive disease and intermittently reached a score of 1 (when APC was <100 G/L).^8^ There was no history of primary or secondary renal dysfunction or hypertension.

### 2.2 Pregnancy history

After repeated unsuccessful attempts to conceive pregnancy, her first healthy child was born full‐term by caesarean section following in vitro fertilization in 2021. Except for an inconsequential singular vaginal blood discharge during the early phase of pregnancy, there was no relevant bleeding in the patient's history. Due to the blood discharge, the preexisting low‐grade thrombocytopenia, and the absence of clinically manifest antiphospholipid syndrome with spontaneously normalized LAC, it was decided to refrain from gestational aspirin prophylaxis. Neither prenatal, perinatal nor postnatal were there any relevant maternal or fetal adverse events, especially no SLE flare or proteinuria.

### 2.3 Current visit

On the current examination, she reported feeling well, and the physical examination was unremarkable. Urine testing showed significant proteinuria and A3 albuminuria in the protein‐/albumin‐to‐creatinine ratio (uPCR 724 mg/g, uACR 488 mg/g). IgG/creatinine was markedly elevated (74.5 mg/g), and α1‐microglobulin was normal, indicating nonselective glomerular proteinuria without tubular impairment (Table 1). APC was stable (108 G/L). There were no other abnormalities suggestive of SLE activity and no signs of hypertension or peripheral edema. The patient recounted no current or desired pregnancy but disclosed that she was overdue for her period after having experienced vaginal spotting 1 week earlier. Prior to initiating an ACE‐inhibitor as initial symptomatic treatment for etiologically unexplained proteinuria, a pregnancy test was performed and was positive. Over the ensuing days, the β‐HCG level increased insufficiently. Detection of a live embryo via ultrasound was not successful, but the intrauterine presence of two amniotic cavities was suspected. Consequently, diagnosis of an early incomplete miscarriage was made, and gestational age was calculated to be 6 + 5 weeks. After experiencing another period‐like vaginal bleeding with tissue shedding 1 week later (7 + 2 weeks), a subsequent ultrasound failed to depict amniotic cavities. Two days later (7 + 4 weeks) a planned suckling curettage was performed. One week later, she had a final vaginal discharge. At this time the urine showed a decrease in proteinuria by over 50% (uPCR 317 mg/g, uACR 210 mg/g, IgG/creatinine 26.9 mg/g). Three weeks later, urine protein was completely normalized, which proved stable in a second control 12 weeks after initial diagnosis. Throughout the follow‐up period, she did not show any SLE relapse.

**TABLE 1:** “Current visit” declares visit of interest.

Lab test	Range/unit	Prior visit	Current visit	First control (q3 weeks)	Second control (q12 weeks)
Serum					
APC	176–391 G/L	>82 (agglut.)	108	113	106
LAC	n.d.	n.d.	n.d.	n.d.	
C3	0.9–1.8 G/L	1	0.92	0.93	0.90
C4	0.1–0.4 G/L	0.19	0.14	0.15	0.15
ANAs (IF)	<1:100			1:6400	
dsDNA‐Ab (ELISA)	<100 IE/mL	22	23	23	
Urine					
uPCR	<100 mg/g	Below LLD	724	317	Below LLD
uACR	<20 mg/g	<9.3	488	210	Below LLD
IgG/creatinine	<12 mg/g		74.5	26.9	Below LLD
α1Mg	<5.0 mg/L		<5.0	<5.0	<5.0

*Note*: Creatinine, cystatine C, GFR, CRP, ESR, urine erythrocytes, and leucocytes were normal.

Abbreviations: Ab, antibody; agglut., pre‐analytic agglutination of platelets; APC, absolute platelet count; IF, immunofluorescence; LAC, lupus anticoagulant; LLD, lower limit of detection (of protein in urine, therefore a ratio could not be calculated); n.d., not detectable; uP‐/uACR, urine protein‐/albumine‐to‐creatinine ratio, α1Mg, α1‐microglobuline; abnormal results are underlined.

## 3 DISCUSSION

In evaluation of proteinuria, there are differential diagnoses that need to be considered in pregnant women. Urinary tract infection (UTI) was ruled out by repeated sterile urine sediment. Urine contamination (with uterine tissue) is no known cause of glomerular proteinuria and therefore highly improbable concerning the reproducible glomerular pattern and gradual (rather than sudden) decrease of urine protein. After ruling out more likely explanations, the 2019 European League Against Rheumatism/European Renal Association–European Dialysis and Transplant Association (EULAR/ERA–EDTA) guidelines recommend a kidney biopsy in patients with at least 500 mg/g uPCR even in the absence of nephritic urine sediment or acute kidney injury.^5^ Recently, observations were confirmed that a large proportion of patients with uPCR <1 g/g had histology of proliferative or membranous LN, whereas a relevant proportion had inactive sediment or normal serology like our patient.^4^, ^9^ Hence, even apparently “inactive” disease may require diagnostic and therapeutic intervention, and proteinuria in our patient could be a manifestation of SLE activity. This requires special attention, as SLE flares in pregnant women are known to manifest as active LN.^7^, ^10^ Nonetheless, the spontaneous remission and absence of clinical symptoms makes this rather unlikely. Before evaluating kidney biopsy in our patient, her pregnancy test was positive, and stagnating β‐HCG indicated impaired pregnancy.

SLE is associated with numerous adverse pregnancy events like preeclampsia and spontaneous abortion that correlate with disease activity.^7^, ^11^, ^12^ While preeclampsia is a known cause of severe proteinuria in pregnancy, it almost exclusively occurs in late pregnancy (>20 weeks of gestation). Gestational age in our case was calculated to be 4 + 6 weeks when proteinuria was first diagnosed. Because of this, and the absence of other indicative symptoms, preeclampsia could be ruled out.

Clowse et al.^13^ postulated the acronym PATH (proteinuria, antiphospholipid syndrome, thrombocytopenia <150 G/L, hypertension) for first‐trimester predictors of early pregnancy loss in SLE patients. Our patient exhibited two of four risk factors. In this regard, preexisting proteinuria could theoretically be placed at the other end of the causal chain.

It therefore remains ambiguous whether the proteinuria was caused by the miscarriage and reflects an “aftermath” of impaired pregnancy or the proteinuria itself was a causative factor for the fetal loss. It is however noteworthy that, during her preceding visit 3 months earlier, she exhibited normal protein excretion.

**FIGURE 1:**
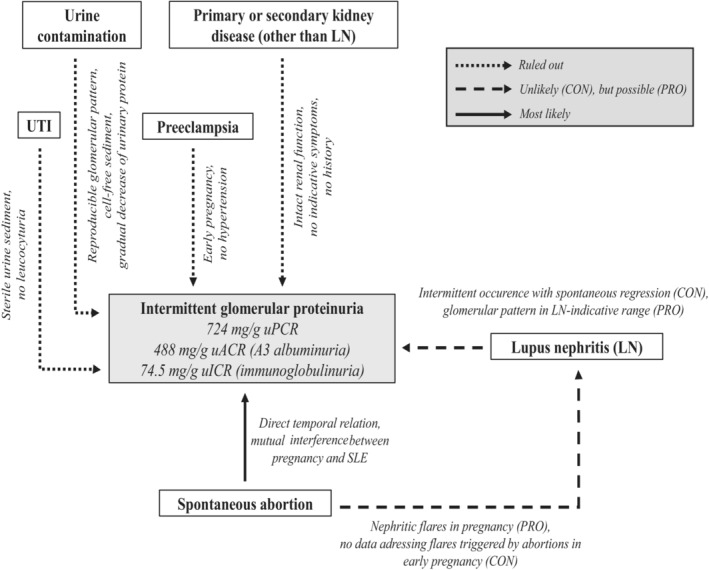
Diagnostic algorithm of intermittent glomerular proteinuria. Clustering in probability of etiology. Arrow labeling indicates diagnostic steps leading to “rule‐out” or “rule‐in” of differential diagnosis. uP‐/uA‐/uICR, urine protein‐/albumine‐/immunoglobulin‐to‐creatinine ratio; UTI, urinary tract infection.

In conclusion, after excluding differential diagnoses (Figure 1), causality of the miscarriage with the urinary findings appears evident. To date, there have been no reports of the concomitant occurrence of early pregnancy miscarriage, possibly LN‐indicative glomerular proteinuria, and its spontaneous regression in a clinically and serologically inactive SLE patient. As the kidney is the organ most affected in SLE pregnancy and the consequences may be very different from persistent proteinuria as would be expected in nephritis, awareness of intermittent proteinuria is crucial.

## AUTHOR CONTRIBUTIONS

**Fabian T. H. Ullrich:** Conceptualization; investigation; visualization; writing – original draft; writing – review and editing. **Alla Skapenko:** Supervision; writing – review and editing. **Hendrik Schulze‐Koops:** Data curation; project administration; supervision; writing – review and editing.

## FUNDING INFORMATION

None.

## CONFLICT OF INTEREST STATEMENT

The authors declare that there is no conflict of interest.

## CONSENT

Written informed consent from the patient was obtained before manuscript submission.

## Data Availability

The data that support the findings of this study are available on reasonable request from the corresponding author. The data are not publicly available due to privacy or ethical restrictions.
